# Assessing the impact of COVID-19 management on the workload of human resources working in India’s National Tuberculosis Elimination Program

**DOI:** 10.1186/s12913-024-11131-8

**Published:** 2024-08-07

**Authors:** Christina Mergenthaler, Aarushi Bhatnagar, Di Dong, Vimal Kumar, Chantale Lakis, Ronald Mutasa, Shankar Dapkekar, Agrima Sahore, Sapna Surendran, Gyorgy Fritsche, Kuldeep Singh Sachdeva, Marjolein Dieleman

**Affiliations:** 1 KIT Royal Tropical Institute, Amsterdam, The Netherlands; 2 The World Bank Group, Washington D.C, USA; 3 Oxford Policy Management, New Delhi, India; 4 The International Union Against Tuberculosis and Lung Disease (South East Asia), New Delhi, India

**Keywords:** Human resources for health, COVID-19, Tuberculosis

## Abstract

**Background:**

In 1993, WHO declared tuberculosis (TB) as a global health emergency considering 10 million people are battling TB, of which 30% are undiagnosed annually. In 2020 the COVID-19 pandemic took an unprecedented toll on health systems in every country. Public health staff already engaged in TB control and numerous other departments were additionally tasked with managing COVID-19, stretching human resource (HR) capacity beyond its limits. As part of an assessment of HR involved in TB control in India, The World Bank Group and partners conducted an analysis of the impact of COVID-19 on TB human resources for health (HRH) workloads, with the objective of describing the extent to which TB-related activities could be fulfilled and hypothesizing on future HR requirements to meet those needs.

**Methods:**

The study team conducted a Workload Indicators and Staffing Needs (WISN) analysis according to standard WHO methodology to classify the workloads of priority cadres directly or indirectly involved in TB control activities as over-, adequately or under-worked, in 18 districts across seven states in India. Data collection was done via telephone interviews, and questions were added regarding the proportion of time dedicated to COVID-19 related tasks. We carried out quantitative analysis to describe the time allocated to COVID-19 which otherwise would have been spent on TB activities. We also conducted key informant interviews (KII) with key TB program staff about HRH planning and task-shifting from TB to COVID-19.

**Results:**

Workload data were collected from 377 respondents working in or together with India’s Central TB Division (CTD). 73% of all respondents (*n* = 270) reported carrying out COVID-19 tasks. The average time spent on COVID-19 tasks was 4 h / day (*n* = 72 respondents). Multiple cadres highly instrumental in TB screening and diagnosis, in particular community outreach (ASHA) workers and CBNAAT/TrueNAAT laboratory technicians working at peripheral, block and district levels, were overworked, and spending more than 50% of their time on COVID-19 tasks, reducing time for TB case-finding. Qualitative interviews with laboratory technicians revealed that PCR machines previously used for TB testing were repurposed for COVID-19 testing.

**Conclusions:**

The devastating impact of COVID-19 on HR capacity to conduct TB case-finding in India, as in other settings, cannot be overstated. Our findings provide clear evidence that NTEP human resources did not have time or essential material resources to carry out TB tasks during the COVID pandemic without doing substantial overtime and/or compromising on TB service delivery. To minimize disruptions to routine health services such as TB amidst future emerging infectious diseases, we would do well, during periods of relative calm and stability, to strategically map out how HRH lab staff, public health resources, such as India’s Health and Wellness Centers and public health cadre, and public-private sector collaboration can most optimally absorb shocks to the health system.

## Introduction

In the past three centuries, tuberculosis (TB) has been declared a pandemic more than once [1, 2]. It is the leading cause of mortality from chronic infectious diseases and is estimated to cause around 4000 mortalities per day globally [1, 3–6]. Individuals infected with TB could transmit the disease to 10–15 people annually [7, 8]. In 1993, WHO declared TB a global health emergency considering 10 million people are battling TB annually, of which 30% were undiagnosed, but it was only in 2018 that TB was labeled as a global priority and achieving a TB-free world was highlighted as a realistic target [2, 9, 10]. A global commitment was made to improve active case finding, implement prevention strategies and strengthen the research agenda, after which TB incidence and mortality declined substantially [10]). However since the start of the COVID-19 pandemic, TB case notifications dropped by 25% globally and TB related mortality increased by an estimated 0.2 to 0.4 million ( [11]. In one year, the COVID-19 pandemic reversed the global progress achieved in the reduction of TB [4, 11].

India has the largest burden of TB globally, with one quarter of the total and one-third of the drug resistant TB burden [6, 12–16]. India has fought to ensure access to appropriate and improved diagnostics and quality of care particularly in the private sector which, among other factors, contributes to an extremely high case fatality rate (an estimated two deaths every three minutes) [7, 16, 17]. When the COVID-19 pandemic hit India, efforts toward TB reduction and control were seriously disrupted and resources were redirected toward the pandemic.

This was a global problem: a survey done in more than 100 countries demonstrated that 78% of TB control programs were disrupted due to COVID-19 pandemic [18–20]. Lockdown, cancellation of essential health services, shifting of human and diagnostic resources from TB to COVID-19 management, and disruption in TB treatment availability impacted TB services at all levels and in the public and private sector [1, 6, 17, 18, 20–23]. The World Health Organization (WHO) estimated a decrease of 18% in reported TB cases in 2020 compared to 2019 with India being a large contributor to that decrease [24]. In India, TB notifications in April 2020 were 78% lower than April 2019, with a larger decline in the private sector [16, 18].

While preventive measures for both diseases are similar (cough etiquette, social distancing and wearing masks), the COVID-19 measures of lockdown, quarantine and redirection of health services delayed TB patient access to diagnosis and treatment [4, 10, 13, 16, 25]. Fear of COVID-19 and imposed pandemic control restrictions led to migration, loss of employment (estimated at 140 million Indians) and increased malnutrition, thus leaving a large number of individuals at risk of both diseases [3, 13, 14]. A study of co-infection of TB and COVID-19 found a 12.3% mortality rate, which is much higher than for only COVID-19 [12].

The health care workforce was and continues to be the main player in the fight against COVID-19. In India as elsewhere, staff and diagnostic equipment (CBNAAT, TrueNaat and GeneXpert) were repurposed to support COVID-19 activities, while research and funds were deprioritized from their initial mandate [1–3, 6, 12, 16, 17, 21–23, 26]. Digital tools and technologies initially created for TB were diverted to the COVID-19 response [20].

Prior to the onset of the COVID-19 pandemic in India in early 2020, India’s National TB Elimination Program (NTEP) finalized its 2021–2025 National Strategic Plan (NSP), which set ambitious TB notification targets. Achievement of these targets cannot be met without proportionately larger gains in numbers of individuals screened, tested and diagnosed with TB, all of which require substantial health workforce investments. India’s Central Tuberculosis Division (CTD), National Health System Resource Center (NHSRC) and the World Bank carried out a Workload Indicators and Staffing Needs (WISN) assessment amongst human resources for health (HRH) involved in public sector TB service delivery, with the objective of projecting future staffing needs to support the NTEP’s NSP and Sustainable Development Goal (SDG) targets for TB notifications by 2025 and 2030. At the onset of the COVID-19 pandemic, when the WISN analysis was conducted, the burden of COVID-19 management fell squarely on the shoulders of the NTEP. Therefore, the aims of this study were to:

1. quantify and qualitatively describe the additional workload imparted by the COVID-19 pandemic on NTEP staff, and;
2. investigate whether there are sufficient hours in a standard workday and work week for cadres heavily involved in TB case-finding to fulfill all TB, non-TB, and COVID-19 requirements.

In this paper we describe the double burden of TB and COVID-19 service delivery amongst HRH working from community to state level in the NTEP and implications of these findings for realistic staffing plans to support achievement of strategic TB control targets.

## Methods

### Study design and population

**Fig. 1:**
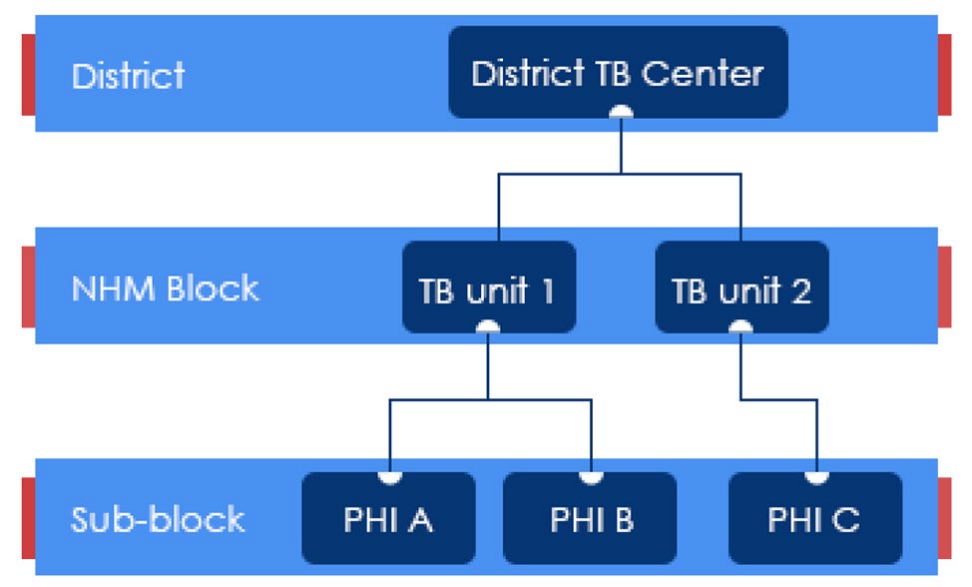
Organogram of peripheral health institutes and TB units reporting to a District TB Center

The study team implemented a convergent parallel mixed methods study design, in which quantitative and qualitative data collection were conducted simultaneously to accommodate project timelines [27]. To accommodate interviewing during the COVID-19 pandemic, the study team conducted an adapted WISN analysis according to standard WHO methodology, as well as the qualitative interviews, by phone. Interviews were designed to quantify the workloads of 28 priority cadres directly or indirectly involved in public sector TB control activities for the year of 2019 (to quantify non-pandemic period workloads), and to project future staffing requirements based on NTEP TB notification targets [28]. In consultation with CTD, NHSRC, and World Bank stakeholders, 18 cadres were selected among the 28 priority cadres for in-depth key informant interviews (KIIs) which were assessed as having relatively greater involvement in managing or providing TB services. State, district, TB unit and peripheral health institute (PHI) level cadres were interviewed for both the quantitative (WISN) analysis and qualitative data collection [29] (Fig. 1).

The study was carried out in 18 districts across seven states of India. These states were purposively sampled to achieve a representative geographic country selection, a representative sample of India’s TB epidemiological transition, as well as representative levels of staff shortage. No additional inclusion or exclusion criteria were applied beyond these three criteria at the state level. Among the selected seven states, districts were stratified based on the NTEP’s TB Index Rank [30] into high, medium and low performance districts [31]. On this basis one district was randomly sampled from each of the high and medium strata for the three smaller states Assam, Himachal Pradesh, Mizoram for a total of six districts. For the four larger states Karnataka, Maharashtra, Tamil Nadu, and Uttar Pradesh, one district was randomly selected from each of the high, medium and low performance districts for a total of 12 districts. This totaled to 18 districts for all seven states. For block and PHI-level cadres, one block per district and one PHI per block were then randomly selected for all cadre interviews. There were a total of 455 NTEP staff working in all 28 priority cadres of the 18 districts for the quantitative study, and 96 among the 18 cadres for the qualitative study. These final sample sizes of 455 individuals for the WISN analysis and 96 individuals for the KII’s were not powered for statistical testing as this was not required for our research aims.

### Tool development

Structured interview tools were prepared to extract necessary data to complete WISN analyses for TB and non-TB activities conducted in 2019, for each of the 28 priority cadres, and then were refined through iterative reviews by HRH and TB experts in India. Additionally, the tools included a module to capture a rough estimate of hours worked on COVID-19 tasks in 2020, along with a description of the tasks. Semi-structured interview guides were developed to steer interviews with key program staff to understand HRH planning and priority shifting before and during the COVID-19 pandemic. WISN tools were piloted amongst 10 staff working in 4 of the 28 priority cadres, and qualitative guides were piloted amongst four staff, each from a different cadre.

### Data collection

All quantitative and qualitative data collection was done in September and October 2020. 393 of 455 eligible TB staff for the WISN (quantitative) analysis were reached by telephone, of which 377 agreed to participate by responding to the WISN questionnaire over the phone, and of which 372 complete questionnaires were obtained. Of the 96 eligible staff for the qualitative KII’s, 60 individuals were reached and participated. Responses were audio recorded and then digitally captured daily in a Microsoft Excel database. Qualitative data collected were captured by recording interviews, transcribing and thematically coding them in Hindi in Microsoft Excel. They were then translated into English. Transcriptions were performed independently by two researchers, then compared and adjusted.

### Data analysis

We carried out quantitative analysis of WISN interview data according to standard WHO methodology [28]. All analysis of WISN data was conducted in Stata Version 16 MP. Workload was calculated at both the individual level and cadre level. A WISN ratio below 0.9 was classified as reflecting low pressure (‘underwork’), between 0.9 and 1.1 as adequate pressure, and above 1.1 as high workload pressure (‘overwork’). The WISN workload analysis and ratios did not factor in COVID-related tasks; therefore, hours reported for COVID-19 are additional and should be considered separately from the number of hours required to fulfill tasks, and staffing projections.

For ten cadres (translating to 225 of 372 respondents) directly involved in TB case-finding activities through either screening or diagnosis, we carried out quantitative analysis in Microsoft Excel to calculate time spent on COVID-19 related tasks on a weekly basis. Average weekly hours spent on TB and non-TB tasks for 2019, and COVID-19 related tasks in 2020 were summed to quantify average weekly workload per cadre required to offer uninterrupted TB services and to additionally manage COVID-19 responsibilities. We applied a framework analysis approach informed by Sousa’s Health Labor Market Framework which served as the conceptual model for our study, to classify, deductively code and interpret the qualitative data from interviews [32]. The methods applied did not account for external factors, including the impact of COVID-19 tasks on workload.

### Ethical considerations

Verbal informed consent was obtained from all participants responding to the WISN questionnaire and participating in key informant interviews, and all participants confirmed that they were in a legal, safe, and convenient environment while conducting the telephonic interview. Ethical approval for all methods including the informed consent process was obtained from Sigma IRB, a division of Sigma Research and Consulting Private Ltd with approval code 10,032/IRB/20–21.

## Results

### Workload pressure and staff shortage

**Fig. 2:**
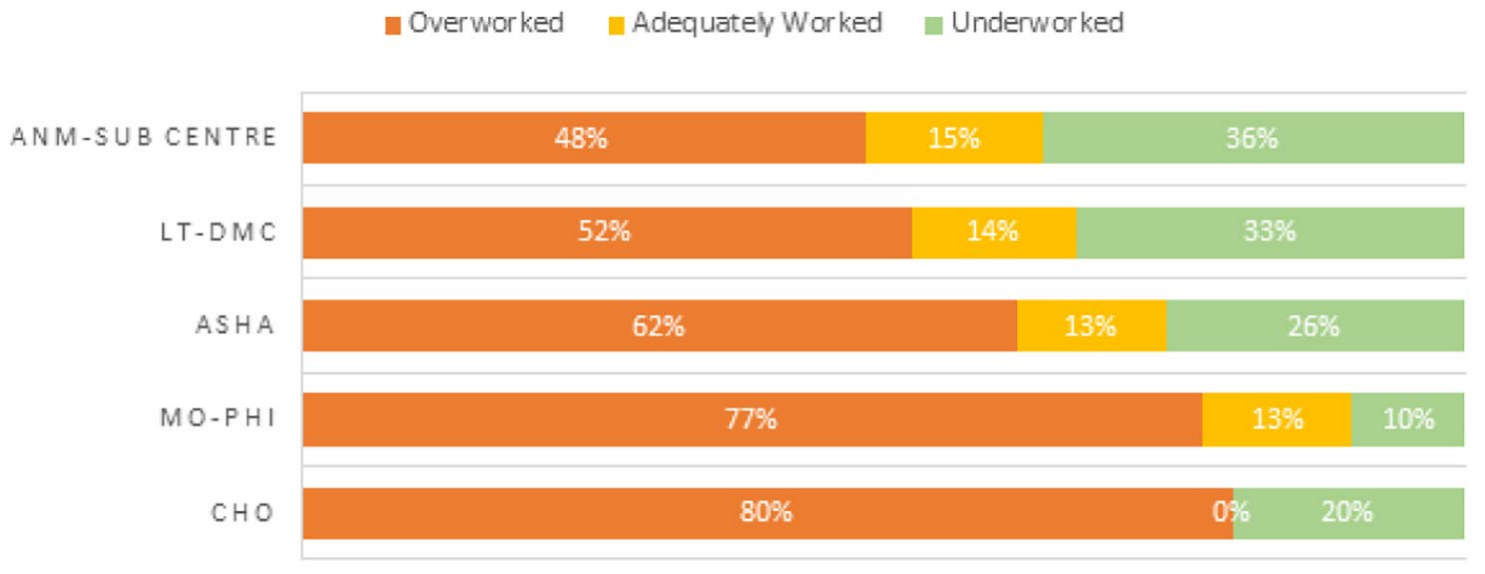
WISN workload pressure by cadre level. PHI level cadres. Data in Figs. 2–5 do not account for time spent on COVID-19 task

**Fig. 3:**
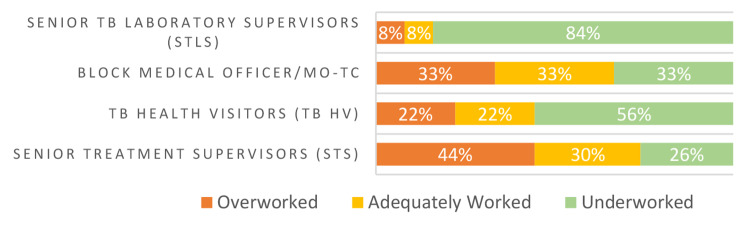
WISN workload pressure by cadre level. TB unit/ block level cadres. Data in Figs. 2–5 do not account for time spent on COVID-19 task

Among 377 interviewed NTEP respondents, a complete WISN workload assessment, not factoring in time spent on COVID-19 tasks, was conducted for 372 respondents. Four out of five cadres in the peripheral health institute (PHI) level were on average overworked, as more than 50% of these cadres’ respondents had a high workload pressure (Fig. 2). All five PHI-level cadres however had an overall staff shortage of 36% (Table 1). At the block level, about one-third of medical officers and senior treatment supervisors (STS) were considered underworked, while most senior TB laboratory supervisors (84%) and approximately half of TB health visitors (56%) were considered underworked. 44% of STS and 33% of medical officers were overworked (Fig. 3).

**Fig. 4:**
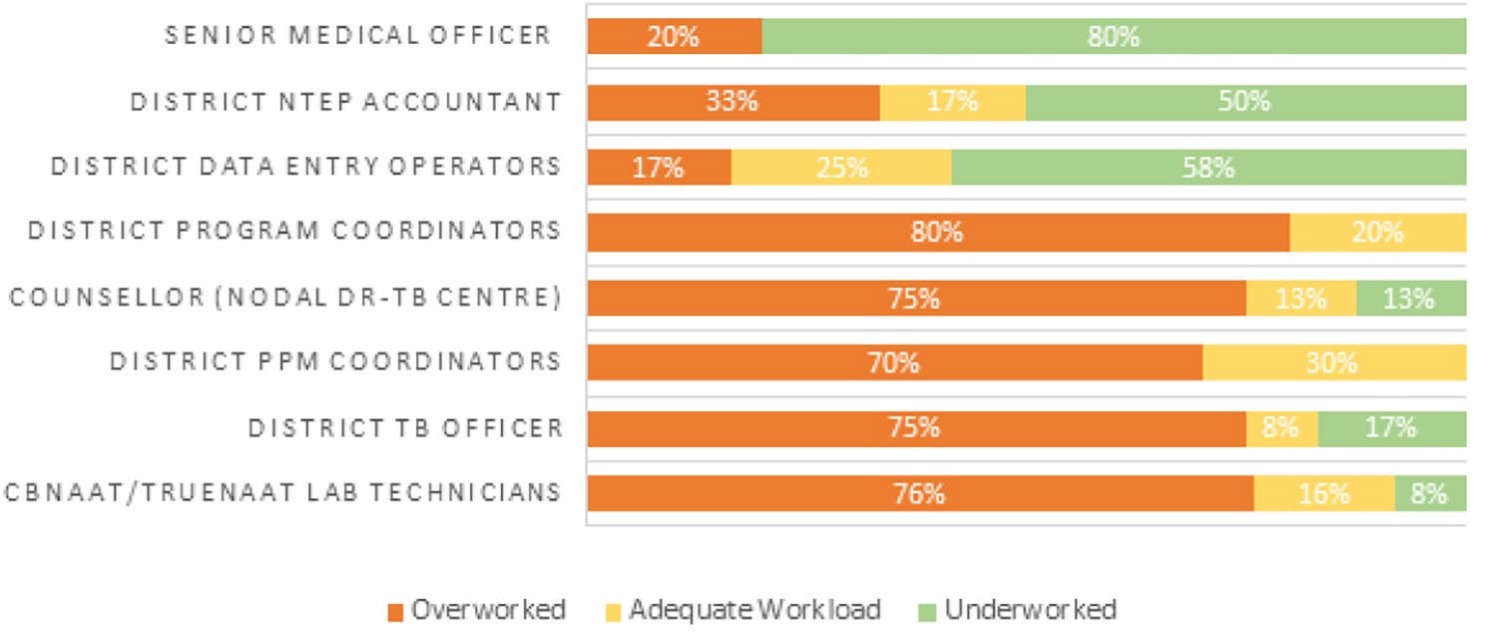
WISN workload pressure by cadre level. District level cadres. Data in Figs. 2–5 do not account for time spent on COVID-19 task

**Fig. 5:**
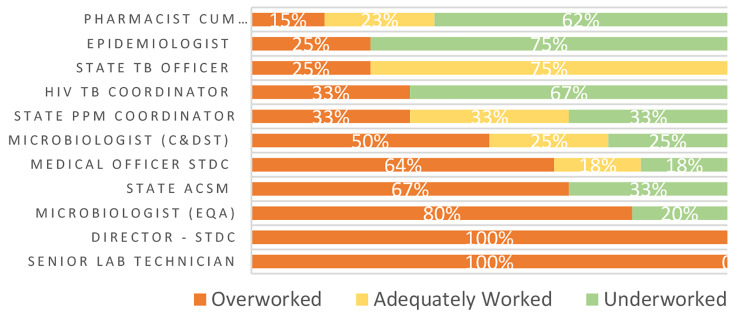
WISN workload pressure by cadre level. State level cadres. Data in Figs. 2–5 do not account for time spent on COVID-19 task

At the district level, five of eight cadres were on average overworked (ranging between 70 and 80% per cadre), with only senior medical officers, NTEP accountants and data entry operators on average being underworked (Fig. 4). Workload showed the widest range at the state level, with pharmacists showing the lowest levels of overwork (15%) and 100% of STDC directors and senior lab technicians reporting overwork (Fig. 5).

District and state level cadres both had an overall shortage of staff (18% each), with only three of eight district level cadres reporting a majority low or adequate workload pressure and surplus of staff, and five of 11 state level cadres reporting the same (Table 1).

**Table 1:** WISN-projected versus current staff available for 28 priority cadres excluding COVID-19 related tasks

Cadres	Staff surveyed (sample size)	Required staff number (based on WISN)	Gap	Workforce Status	WISN ratio	Workload pressure
**Peripheral Health Institute**						
DMC LT	21	23	2	Shortage	1.07	Adequate
ANM Sub-Centre	33	41	8	Shortage	1.24	High
CHO	5	7	2	Shortage	1.40	High
MO PHI	31	47	16	Shortage	1.52	High
ASHA	39	84	45	Shortage	2.15	High
**PHI sub-total**	**129**	**202**	**73**	Shortage (36%)		
Block / TB Unit						
Senior TB Laboratory Supervisors	25	17	-8	Surplus	0.76	Low
Block Medical Officer	24	25	1	Shortage	1.08	Adequate
TB Health Visitors	9	11	2	Shortage	1.16	High
Senior Treatment Supervisors	27	32	5	Shortage	1.29	High
**Block sub-total**	85	85	0	Adequate		
District						
Senior Medical Officer	10	7	-3	Surplus	0.68	Low
District NTEP Accountant	12	10	-2	Surplus	0.87	Low
Data Entry Operator	12	11	-1	Surplus	0.91	Adequate
District Programme Coordinators	5	6	1	Shortage	1.20	High
Counsellor (DR-TB)	8	11	3	Shortage	1.32	High
District PPM	10	14	4	Shortage	1.36	High
District TB Officers	12	17	5	Shortage	1.40	High
CBNAAT/True NAAT	25	39	14	Shortage	1.58	High
**District sub-total**	**94**	**115**	**21**	Shortage (18%)		
**State**						
TB-HIV Coordinator	3	2	-1	Surplus	0.77	Low
Epidemiologist	4	3	-1	Surplus	0.86	Low
Pharmacist - Storekeeper	13	13	0	Optimal	1.01	Adequate
State ACSM	6	6	0	Optimal	1.05	Adequate
State PPM Coordinator	3	3	0	Optimal	1.09	Adequate
Microbiologist (IRL)	4	5	1	Shortage	1.20	High
Medical Officer	11	13	2	Shortage	1.21	High
State TB Officer	4	5	1	Shortage	1.24	High
Microbiologist (EQA)	5	7	2	Shortage	1.43	High
Director STDC	4	7	3	Shortage	1.65	High
Senior Lab Technician	7	14	7	Shortage	2.04	High
**State sub-total**	**64**	**78**	**14**	Shortage (18%)		

### Double workload burden: COVID-19 and Tuberculosis

Respondents from all 28 cadres carried out COVID-19 tasks daily, approximately 73% of 372 total respondents (*n* = 270) [33]. The average time spent on COVID-19 tasks was 4.4 h per day, with PHI and block level cadres reporting the highest average of 5.4 and 5.0 h per day respectively, followed by state at 4.2 h and district at 3.9 h per day [33]. This also holds true for multiple cadres which are both highly instrumental in TB screening and diagnosis and already overworked according to the WISN analysis who *did not* factor in the time required for their additional COVID-19 tasks.

Table 2 presents an overview of the time spent on reported COVID-19 (2020), TB (2019), and non-TB related activities (2019) on a weekly basis for ten cadres which have key roles in either screening or testing for TB. This means that each of these cadres is directly involved in identification of people with presumptive TB in the community or facility, or testing specimens for TB in the laboratory (column C). Relative to a 40-hour workweek, all 10 cadres reported that they spent more than 50% of their daily hours (extrapolated to the week) on COVID-19-related tasks (column L). Seven of the ten cadres are already overworked (columns E, F) without factoring in COVID-19 responsibilities. Summing the COVID-19, TB, and non-TB hours required, these cadres would need to work an average of 1.7 (range: 1.5 to 1.8) 40-hour work week equivalents per week, for an average of 66.8 h per week (range: 60.0–72.0). The hours spent on TB activities (column I) which are spent specifically on case-finding activities (column J) are also presented to provide a perspective on the number of hours available for work directly related to identifying, screening and testing people for TB. On average these cadres spend 32.9% of their week (26.9 h) on case-finding activities, if they have no additional COVID-19 responsibilities.

**Table 2:** Impact of COVID-19 on workload of cadres heavily involved in tb screening & diagnosis

A	B	C	D	E	F	G	H	I	J	K	L	M	*N*
Level	Key screening & diagnosis cadres	Case-finding activity	Sample size	Mean WISN ratio	Workload pressure	% time/ week on TB	% time / week on TB case-finding (CF)	TB hours / week	TB hours / week (spent on CF)	Non-TB hours/ week	COVID hours / week	TB, non-TB & COVID hours/ week	Required / available weekly hours
Peripheral health institute	ASHA	Patient referral & community screening	39	2.15	High	38.7%	19%	15.48	7.6	24.52	23.5	63.5	**1.6**
	MO-PHI	Attending presumptive TB patients at OPD	31	1.52	High	21.5%	5%	8.6	2	31.4	30.5	70.5	1.8
	LT-DMC	Performing sputum smear microscopy & NAAT testing	21	1.07	Adequate	62.0%	55%	24.8	22	15.2	26.0	66.0	1.7
	Community Health Officer	Providing OPD services	5	1.4	High	*85.0%*	41%	34	16.4	6	30.0	70.0	**1.8**
	Auxiliary nurse-midwife	Supporting medical officer in TB related OPD	33	1.24	High	*77.0%*	12%	30.8	4.8	9.2	25.0	65.0	**1.6**
Block	TB Health Visitor	Active case-finding	9	1.16	High	*98.0%*	14%	39.2	5.6	0.8	20.0	60.0	**1.5**
	Senior treatment supervisor	Active case finding	27	1.29	High	*89.0%*	17%	35.6	6.8	4.4	25.0	65.0	**1.6**
	Senior TB Laboratory Supervisor (STLS)	Oversight lab activities	25	0.67	Low	85.9%	0%	34.36	0	5.64	29.0	69.0	1.7
District	CBNAAT/True NAAT Lab Technicians	CBNAAT testing	25	1.58	High	88.0%	100%	35.2	40	4.8	32.0	72.0	1.8
	Senior Medical Officer	oversight of TB activities	10	0.68	Low	65.4%	0%	26.16	0	13.84	27.0	67.0	1.7
Total						67.4%	32.9%*	26.9	13.2*	13.1	26.8	66.8	1.7

*excludes cadres reportedly spending zero hours on direct case finding activities

Columns E – L = derived from WISN analysis (J is a subset of I); M = sum of columns I, K, L; N = column M / 40 h

### Qualitative findings

Interviews with NTEP staff revealed that cadres at all levels were responsible for setting up COVID-19 testing labs; ensuring that proper equipment for health care workers and frontline staff were provided and distributed; organizing COVID-19 specimen collection, transportation, testing and results provision. These COVID-related tasks increased workload primarily of community and PHI level cadres, in effect compromising NTEP service delivery in screening and diagnostic activities, supervision, and monitoring. Many lab technicians were diverted from TB to prioritize COVID-19 testing as the same testing platforms (CBNAAT) were shared to process both. TB testing also decreased during the national lockdown, in which all transportation was banned and most businesses were closed. One respondent expanded on this:

“Of course, yes, being involved in work related to COVID management in the district has hampered our work. We’ve had to divert staff for testing and sampling. People were assigned for identifying and following up cases etc., tracking the home isolation cases, so work still continues to suffer. We still don’t have enough to manage, how to handle all the samples? At one time, some 1,500 samples came. So, to get samples diagnosed at the field level, we had to mobilise the lab technicians’.” (CMOH)

Supervisory staff also prioritized COVID-19 related tasks:

“Supervisory staffs have also been given COVID duty and their work also got affected. That is why we have 23% decrease in notification.’ (State TB Officer).”

Respondents observed a large reduction in healthcare seeking behavior due to COVID-19 and hypothesized that this was related to fear of being tested for COVID-19, becoming infected in a facility, or being stigmatized due to ambiguous symptoms. Stakeholders reported that apprehensions around COVID-19 also added to TB stigma- that patients did not want to report symptoms including cough or cold, because they were apprehensive of having to test for COVID-19 and of possible institutional quarantine, as shown in this quote:

“There is stigma among the patient for COVID…they are not willing to come to the health facility, doctors are not willing to see the patients because of fear of contracting of the disease. Probably from October private institutions will start opening and probably things will settle gradually, but it all depends on availability of a remedy for COVID’. (DHS)”

However, responses suggest that the NTEP tried to cope with the additional COVID-19 workload by screening for COVID-19 during routine TB active case finding activities, and reduce interruptions to routine service delivery as much as possible. For instance, PHI and block level cadres conducting COVID-19 awareness campaigns, case-finding, and supporting patients at home and institutional quarantine often tried to integrate these tasks into their routine community-based TB tasks. The following quote provides an example of NTEP staff creating efficiency gains:

“My attempt has been to take advantage of the situation to combine other aspects. There haven’t been problems in getting people medicines. ASHA’s have to do per day surveys on COVID. We suggested that since they have to visit these many houses a day, they should continue their other work including Ante-Natal Care, healthcare for kids, general health, and TB. If you only ask about COVID — do you have fever, a cough and so on — it leads to the public getting scared of testing. So, the ASHA’s have been sensitized to talk about all aspects of their work.’ (District Nodal Officer CP)”

“But we are committed to the goal of TB elimination. We have to do some active case finding activities after this and treat the patients. The STSs are delivering TB drug to the patients at their doorstep who are not able to come to the centers; sputum cups are provided to the ACSM coordinator during community [COVID-19] survey to collect [COVID-19 and TB] sputum samples from the patients .(District TB Officer).”

Providing drugs to TB patients was initially challenging during COVID-19 lockdowns; however, service providers quickly responded by providing extra drugs to patients during their facility visits and by conducting home deliveries:

“When we came into lockdown the policy was made that drugs should be brought to the patient instead of patient approaching us for drugs that is one thing. We also took on the decision that no dropout (of TB patients) should happen for want of drugs or want of medical aid’. (DHS)”

## Discussion

### Summary

The WISN analysis shows that cadres working in all levels of the NTEP were overworked and suffered from staff shortages, which were substantial for both cadres (PHI and block) working closest with the community. Managing COVID-19 has seriously increased the workloads of many of these cadres, and COVID-19 tasks were reportedly prioritized above many others. Qualitative interviews suggest that the workloads calculated may be based on fewer facility attendees than expected due to reduced healthcare seeking behavior and provider hesitance to interact with patients. The WISN analysis and COVID-19 workload calculations suggest that reaching the NTEP’s 2025 notification targets may be challenging if a similar diversion of key TB screening and diagnosis staff towards COVID-19 management were to continue. To accommodate the double burden of TB and COVID-19, NTEP staff implemented workarounds, including simultaneously conducting TB and COVID screening, and delivering medicines in bulk quantity to patients’ homes during community COVID-19 surveillance.

### WISN ratios underrepresent workloads

Due to the omission of COVID-19 tasks from the WISN ratio and the expectation for key cadres to carry out COVID-19 and TB services simultaneously, it can be reasoned that the actual workload pressure experienced in 2020 was likely much higher than presented. As most key screening and diagnostic cadres would have normally spent more than 50% of their weekly hours on TB tasks prior to COVID-19, they were working far beyond 40 h per week, and cutting back on delivery of routine TB services to accommodate the COVID-19 workload [3, 4, 6, 12, 16, 24]. Although lockdowns limited healthcare seeking behavior and reduced TB workload for many, demand for TB services was still present as evidenced by continued high notifications: overall 2.4 million and 1.8 million cases were notified in 2019 and 2020 respectively [34]. Our analysis suggests that for uninterrupted TB service delivery to have co-existed with the volume of COVID-19 activities conducted in 2020, almost twice the number of HRH would have been required just among cadres involved in key TB screening and diagnostic activities (mean: 1.7; range: 1.5–1.8). Thus, in a COVID-19 endemic setting, the cadres who are most heavily involved in presumptive TB case-finding have minimal time to pull presumptive TB cases into the care cascade, upon which all NTEP TB notification targets depend. Passive case-finding of presumptive TB cases in facilities has returned to pre-pandemic levels in many settings, due to health system resilience and international funding mechanisms. However our study shows that cadres responsible for TB active case-finding (ASHAs, TB health visitors and senior treatment supervisors), which is also an essential activity to reach the NTEP’s notification targets, are overworked without COVID-19 tasks, and required at least 1.5–1.6 times their current working hours to provide uninterrupted routine TB and COVID services in 2020. While NTEP cadres can be recruited and retained to address this gap during future health emergencies, this is a particularly problematic finding for ASHAs, who are not employed staff, but volunteers who are paid for performance for a range of community-based services. In fact, ASHAs had the highest WISN ratio (2.15) of all 28 priority cadres interviewed. It is not a sustainable solution to, in emergency situations, rely more heavily on an overworked group of individuals who are not compensated equitably relative to employed NTEP cadres.

### Solutions identified

Our results highlight a number of strategies implemented by NTEP staff to mitigate the impact of COVID-19 responsibilities on routine service delivery, namely adding COVID-19 to existing community outreach services, or bringing TB medications to patient homes during lockdowns. In facility settings, the CTD has implemented bi-directional TB and COVID-19 screening among higher risk groups [35]. Other opportunities to create efficiencies have been well-documented elsewhere [4, 7, 11, 17, 21, 24, 36].

These solutions have and likely will continue to support NTEP cadres, at all levels, in managing both routine TB and COVID-19 responsibilities. However, there are still several obstacles that need to be addressed if these solutions can provide sustained relief to overworked staff. ASHAs have demanded improved compensation and recognition for the important role they have played in carrying out community health outreach, including both TB and COVID-19 case-finding [37].

Furthermore, although the influx of COVID-dedicated PCR machines may have been hugely beneficial, for the foreseeable future India will need to test a high volume of specimens for COVID-19, and a backlog of undiagnosed prevalent TB cases which may have accrued during COVID-19 peaks [4]. This will require both additional PCR machines and laboratory staff. Finally, ensuring that people who test positive for TB or are clinically diagnosed are started on TB treatment will require additional senior treatment supervisors and medical officers.

### Next steps

To make gains toward TB notification targets, supporting the capacity of cadres involved in screening and diagnosis of TB is essential. In addition to providing more appropriate incentives to ASHAs, India’s private sector has a proven track record of conducting TB case-finding and can play a larger role in diagnosis & referral [38]. Within facilities, identifying more TB presumptives will be key, and can be achieved by conducting systematic screening in outpatient departments [39], although this will also require additional capacity for facility-based cadres.

Others have written about the necessity of strategies to reach populations who avoided health services during lockdowns and infection waves [4, 10]. Strengthening this linkage between communities and facilities to increase presumptives to funnel into the TB screening cascade is essential, and may require increased HRH during future public health emergencies [40, 41]. The more recently established Health and Wellness Centers can play an important role in providing both TB and COVID-19 screening services to communities, although this will add supervision responsibilities [42].

Given the mix of differently burdened staff in similar roles working in near proximity, the study identified task-sharing and shifting as a potential solution [33, 43]. This could be explored between NTEP cadres or with India’s recently emerging public health cadres. In 2022 India’s Ministry of Health and Family Welfare (MoHFW) published implementation guidance for public health management cadres with the specific mandate to manage infectious disease outbreaks in health facilities, disease control offices, and educational settings [44, 45]. Tamil Nadu, Maharashtra, Chhattisgarh, West Bengal and Odisha states have already trained and stationed public health management cadres, while other states are yet to do so [46]. The permanent availability of such a cadre could provide needed support for management of newly emerging diseases, such as COVID-19, and minimizing disruption to routine health services in the early stages of future emergencies.

### Study limitations

WISN analyses are intended to be conducted through in-person observation, but due to lockdown restrictions our analysis was conducted on the telephone. Therefore, our findings were based on reported as opposed to observed activity duration, introducing potential recall bias. Furthermore, the study design did not allow for tests of statistical significance to be conducted, nor for comparisons between urban and rural or sector strata.

## Conclusion

The devastating impact of COVID-19 on HRH capacity to conduct TB screening and diagnosis in India in 2020 and 2021, as in other settings, cannot be overstated. Our findings provide clear evidence that NTEP HRH did not have time or essential material resources to carry out TB tasks during the COVID-19 pandemic without doing substantial overtime and/or compromising on TB service delivery. To minimize disruptions to routine health services such as TB amidst future emerging infectious diseases, we would do well, during periods of relative calm and stability, to strategically map out how HRH lab staff, public health resources, such as India’s Health and Wellness Centers and public health cadre, and public-private sector collaboration can most optimally absorb shocks to the health system.

## Data Availability

All data generated or analyzed during this study are available from India’s Central Tuberculosis Division, and may be provided upon reasonable request to one of the co-authors.
